# Supplementary material for: Biallelic variants in RNU2-2 cause a remarkably frequent developmental and epileptic encephalopathy
Source: Nat Genet. 2026 Mar 30;58(4):798–809. doi: 10.1038/s41588-026-02551-9 (PMC13083258; doi:10.1038/s41588-026-02551-9)
Supplement: Supplementary file 1 — Supplementary Figs. 1–4 and Supplementary Note. [file 41588_2026_2551_MOESM1_ESM.pdf]

nature genetics

Supplementary information https://doi.org/10.1038/s41588-026-02551-9

Biallelic variants in *RNU2-2* cause a remarkably frequent developmental and epileptic encephalopathy

In the format provided by the authors and unedited

Supplementary Information

Contents

Supplementary Information 1
Supplementary Figures 3
  Supplementary Figure 1 3
  Supplementary Figure 2 4
  Supplementary Figure 3 5
  Supplementary Figure 4 6
Supplementary Note 7
  Family 4 (n.20G>A) 7
    Proband (Individual 4) 7
    Sibling (Individual 5) 7
  Family 5 (n.20G>A) 8
    Individual 6 8
  Family 53 (n.28C>G) 9
    Individual 52 9
  Family 29 (n.102T>C) 10
    Proband (Individual 36) 10
    Sibling (Individual 37) 10
  Family 6 (n.104T>C) 11
    Proband (Individual 7) 11
    Sibling (Individual 8) 11
    Sibling (Individual 9) 12
  Family 7 (n.116_127del) 13
  Individual 10 13
  Family 8 (n.127G>C) 14
  Individual 11 14
  Family 3 (n.166G>C) 15
    Individual 3 15
  Family 21 16
    Proband (Individual 27, n.5C>T | n.129T>C) 16
    Sibling (Individual 28, n.5C>T | n.*9_*29del) 16
  Family 14 (n.8C>T | n.104T>G) 17
  Individual 17 17
  Family 47 (n.12G>C | n.171T>C) 18
  Individual 57 18

Family 41 (n.18_21delinsGGG | n.45C>T) ... 19
Individual 50 ... 19
Family 35 (n.19G>A | n.52G>A) ... 20
Individual 44 ... 20
Family 27 (n.19G>A | n.63G>A) ... 21
Individual 34 ... 21
Family 49 (n.20G>A |n.21C>A) ... 22
Individual 59 ... 22
Family 48 (n.21C>A | n.180G>A) ... 23
Individual 58 ... 23
Family 28 (n.25G>A | n.131G>C) ... 24
Individual 35 ... 24
Family 17 (n.28G>C | n.158G>C) ... 25
Proband (Individual 20) ... 25
Sibling (Individual 21) ... 25
Family 46 (n.157G>C | n.31G>A) ... 26
Individual 56 ... 26
Family 36 ([n.36G>A ;n.154C>T] | n.182T>C) ... 27
Individual 45 ... 27
Family 26 ([n.43T>G;n.62T>G] | [n.162dup;n.189T>C]) ... 28
Individual 33 ... 28
Family 22 (n.43T>C | n.175G>A) ... 29
Individual 29 ... 29
Family 32 (n.45C>G | n.50C>T) ... 30
Individual 41 ... 30
Family 34 (n.45C>T | [n.67C>T;n.183_184insA]) ... 31
Individual 43 ... 31
Family 42 ([n.56A>T ;n.157G>C] | n.104T>C) ... 32
Individual 51 ... 32
Family 44 (n.104T>C | n.100T>G) ... 33
Individual 53 ... 33
Family 45 (n.104T>C | n.159_176dup) ... 34
Proband (Individual 54) ... 34
Sibling (Individual 55) ... 34
Specific variants and variant combinations ... 35
Supplementary References ... 36

Supplementary Figures

Supplementary Figure 1

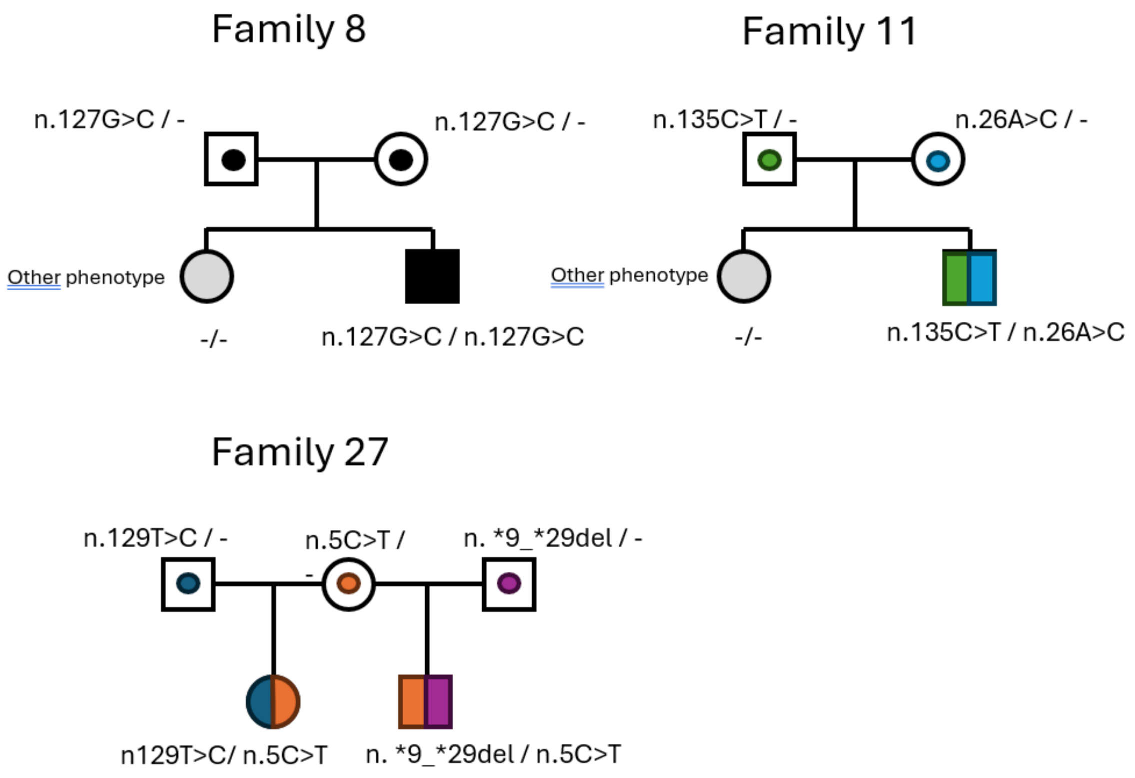

Supplementary Figure 1| Pedigrees of Families 8, 11 and 27 where segregation of variants occurs either with siblings with different phenotypes or with overlapping phenotypes but distinct variants from different fathers.

Supplementary Figure 2

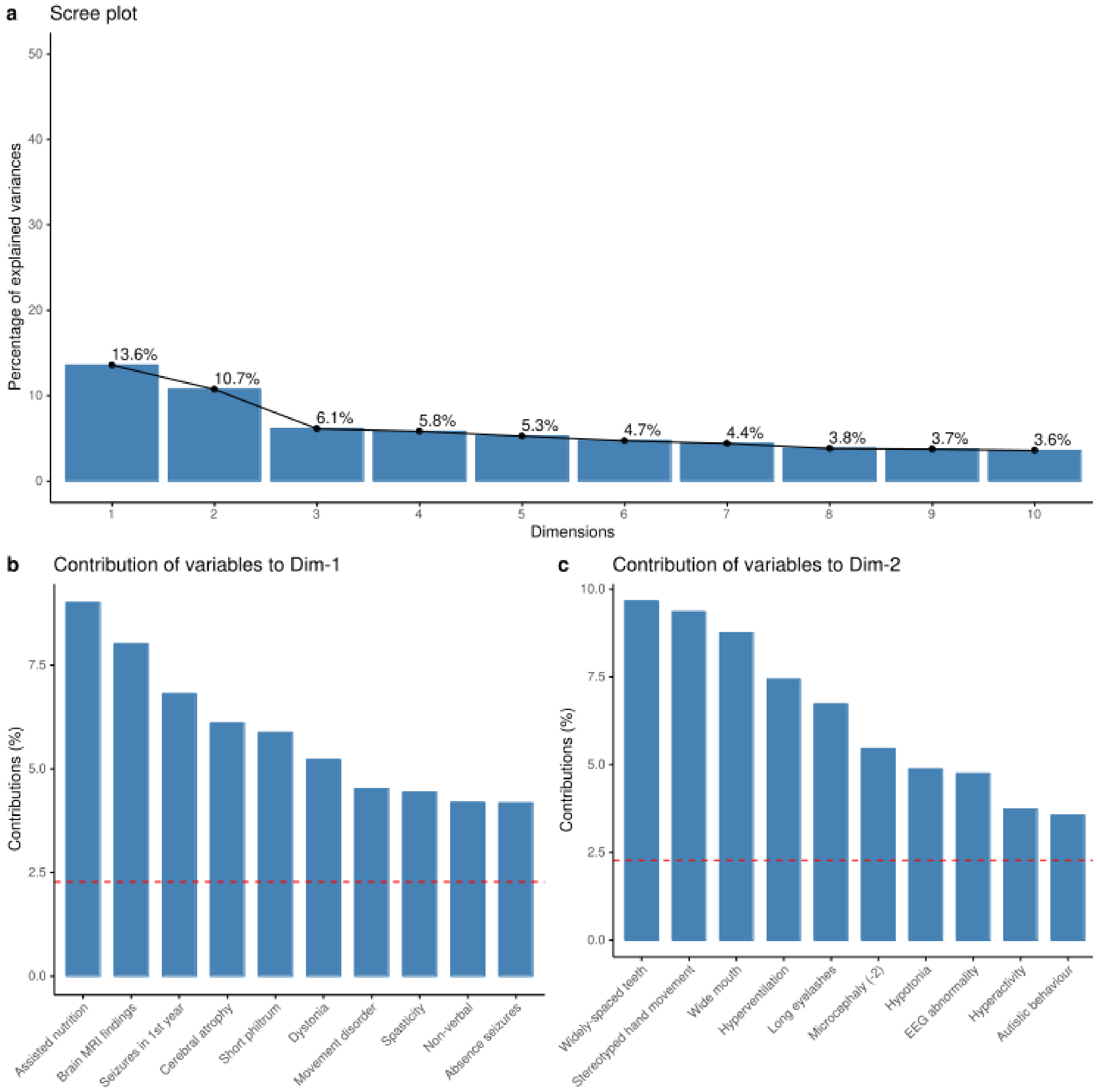

Supplementary Figure 2| Details of the PCA analysis comparing HPO term frequency in the recessive and dominant *RNU2-2* disorders. **A)** Proportion of the variance explained by the first ten principal components. **B)** The relative contribution of the 10 most influential HPO terms to the first principal component. **C)** The relative contribution of the 10 most influential HPO terms to the second principal component. The red dashed line indicates the expected value if each contribution was uniform.

Supplementary Figure 3

```
RNU2-2      ATCGCTTCTCGGCCTTTTGGCTAAGATCAAGTGTAGTATCTGTTCTTATCAGTTTAATAT      60
RNU2-1      ATCGCTTCTCGGCCTTTTGGCTAAGATCAAGTGTAGTATCTGTTCTTATCAGTTTAATAT      60
            ************************************************************

RNU2-2      CTGATACGTCCTCTATCCGAGGACAATATATTAAATGGATTTTTGGAAATAGGAGATGGA      120
RNU2-1      CTGATACGTCCTCTATCCGAGGACAATATATTAAATGGATTTTTGGAGCAGGGAGATGGA      120
            ***********************************************    *********

RNU2-2      ATAGGAGCTTGCTCCGTCCACTCCACGCATCGACCTGGTATTGCAGTACTTCCAGGAACG      180
RNU2-1      ATAGGAGCTTGCTCCGTCCACTCCACGCATCGACCTGGTATTGCAGTACCTCCAGGAACG      180
            ************************************************* **********

RNU2-2      GTGCACTCTCC 191
RNU2-1      GTGCACCC--- 188
            ****** *
```

Supplementary Figure 3| Multiple sequence alignment of RNU2-2 and RNU2-1 genes highlighting divergent nucleotides.

Supplementary Figure 4

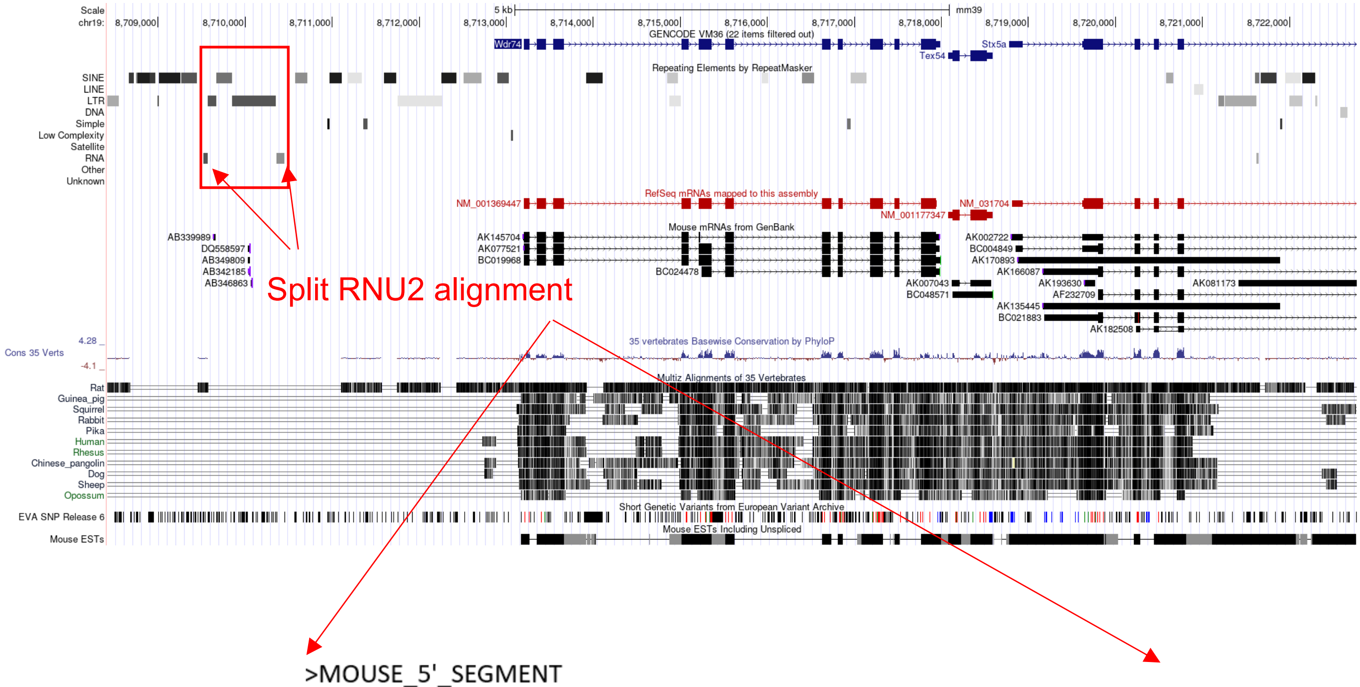

>MOUSE_5'_SEGMENT
----GTTTCTCAGCCTTTGACTAAAATCAAGTGTAGTATTTGTTCTTACTA---------

>HUMAN_RNU2-2P
ATCGCTTCTCGGCCTTTTGGCTAAGATCAAGTGTAGTATCTGTTCTTATCAGTTTAATAT
CTGATACGTCCTCTATCCGAGGACAATATATTAAATGGATTTTTGGAAATAGGAGATGGA
ATAGGAGCTTGCTCCGTCCACTCCACGCATCGACCTGGTATTGCAGTACTTCCAGGAACG
GTGCACTCTCC
>MOUSE_3'_SEGMENT

--TTTGGAATTAGGAGGTAGA
ATGGGAGCTTGCTCTGTCCACTCTATGCACTGATTGGGCATTGCAGTACTTCTGGGAATG
GTGTATC----

Supplementary Figure 4| Truncation of the orthologous RNU2 copy upstream of WDR74 in mice (GRCm39) due to a nested SINE in LTR retrotransposition event. The 5' segment of the RNU2 truncated copy aligns to the 5' region of human RNU2-2 and the 3' segment likewise to the 3' region of human RNU2-2.

Supplementary Note

Family 4 (n.20G>A)

Proband (Individual 4)

This is a female individual of British Asian (Pakistani) background, currently aged 14y. She is the third child born to healthy 2nd cousin parents. She was born at term with no issues in pregnancy. Her early development was normal until she developed infantile spasms at 6 months of age. Following this her development plateaued, and she developed severe epileptic encephalopathy with microcephaly. She presented with infantile spasms which initially settled, then tonic clonic seizures, drop attacks, focal seizures and absence seizures. She had cluster seizures in childhood but no prolonged seizures. Serial EEGs revealed severe epileptic encephalopathy and her MRI scan revealed significant atrophy.

At age 14 years she was non-verbal, non-mobile (she was unable to sit independently), severely microcephalic (HC 4SD below the mean). She had generalised tonic-clonic seizures (worse at night), focal seizures (grimacing and dribbling), and absence seizures. Her epilepsy was managed with multiple antiepileptic medications and a VNS. In addition, she had camptodactyly of her 5th finger, hip problems, scoliosis, flexion contraction of the right knee and constipation.

She was recruited to the 100KGP, however primary analysis did not identify any relevant pathogenic or likely pathogenic variants. Cohort-wide analysis of the 100KGP identified a homozygous n.20G>A variant in RNU2-2.

Sibling (Individual 5)

This is a male individual of British Asian (Pakistani) background, currently aged 10y. He has severe epileptic encephalopathy akin to Lennox-Gastaut syndrome, worsening dyskinetic movement disorder, severe global developmental delay, severe microcephaly and camptodactyly.

He was born at term and observed on the neonatal unit for respiratory distress but did not require treatment. He had low tone compared to his siblings. His initial milestones were normal. His mother noticed subtle problems from 6 months (difficulty with latching, never learned to sit). He developed infantile spasms at 7-8 months of age. EEGs showed hypsarrhythmia. Spasms decreased in frequency with treatment (ACTH, prednisolone, vigabatrin) but still occurred daily. His complex epileptic encephalopathy initially evolved in a similar pattern to his sister (Individual 4) with similar EEGs and MRI findings. He developed absence, tonic, and generalised tonic clonic seizures. At age 2, the seizures worsened (drop attacks, right and left sided body seizures). However, he also developed a florid dyskinetic movement disorder, significantly improved with tetrabenazine. These movements mimicked seizures and included choreoathetoid movements of limbs, geniospasm, lip smacking, protruding tongue. They often proceeded and followed a seizure. At 10 years of age he had severe global delay (unable to sit, non-verbal), severe microcephaly (-3.2SD), camptodactyly, and has had two intensive care admissions with respiratory failure related to secretion clearance.

Along with his sister, he was recruited to 100KGP. No diagnosis was found initially. He was also found to carry the same n.20>A RNU2-2 in homozygous state.

Family 5 (n.20G>A)

Individual 6

This is a male individual of Pakistani background, currently aged 15 years. Pregnancy and perinatal history was unremarkable. He was delivered at term with birth weight 7lbs. Birth OFC was not recorded. At most recent assessment he was noted to be very tall and microcephalic.

His motor development was delayed and he never attained the ability to walk. He is also non-verbal with severe intellectual disability and a diagnosis of Autism spectrum disorder. He has clonic seizures which are controlled with clobazam, levetiracetam and valproate. He is globally hypotonic without spasticity. His MRI brain was reported as normal.

He was recruited to the 100KGP and the primary analysis did not identify any pathogenic variant. Our cohort analysis revealed a homozygous n.20G>A variant in RNU2-2, as was identified in the two siblings from Family 4, although both families are not knowingly related. Haplotype analysis revealed that Individual 6 shares a ~30Mb region of homozygosity encompassing RNU2-2 with P4 and P5. Single nucleotide variant analysis within this region reveals a shared haplotype and hence this likely represents a common ancestral variant rather than a recurrent variant.

Family 53 (n.28C>G)

Individual 52

This is a male individual of Tajik (Afghani) background, currently aged 10 years. Clinical features were first noted at 6 months. Pregnancy and perinatal history were unremarkable. He was delivered at 40 weeks.

At age 10y4m years, height measured 134cm (-1SD). weight measured 32kg (0SD). OFC was 48cm (-4SD). His motor development was delayed, and he rolled over at 10 months and sat unsupported at 12 months. His first steps were at 36 months. He had speech delay and has severe intellectual disability.

Early in life, he was diagnosed with epileptic encephalopathy having both atonic seizures and tonic seizures with impaired awareness. These were previously intractable to lamotrigine, pulsed methylprednisolone, pulsed prednisolone and clobazam. Seizures have improved with Sodium valproate 250 mg bd, Topiramate 50 mg bd and Rufinamide 200 mg bd.

His brain MRI was reported as normal at 6years 10months. He has dysmorphic facial features with prominent supra-orbital ridge, deep-set eyes, medial eyebrow flaring, synophrys, long eyelashes, thick upper vermillion border and everted lower lip. He also has right-sided sensorineural hearing loss at 1000 Hz range only.

Trio genome sequencing, though UDNAus revealed a homozygous n.28C>G variant in RNU2-2. Additional genetic findings included: Extensive long continuous stretches of homozygosity (LCSH) on chromosomes 1, 2, 3, 5, 6, 7, 9, 10, 11, 12, 14, 16, 19, 21 (representing approx 12.1% of the genome), however no other pathogenic or plausible causative candidate variants were identified. Two unaffected siblings did not share a region of LCSH over the 11q12.3 region containing RNU2-2 on SNP microarray.

Family 29 (n.102T>C)

Proband (Individual 36)

This is a female individual of Pakistani background, currently aged 16. Clinical symptoms were first noted at 6 months. Pregnancy and perinatal history were unremarkable. She was delivered at term with a birth weight of 3.4kg. Growth assessment at age 16 years: weight measured 43kg and OFC was 51 cm (-.028).

Developmental history was notable for motor delay. She was never able to roll over, has never sat unsupported or walked. She also is non-verbal with profound intellectual disability.

Neurological findings were remarkable for seizures, which are now tonic-clonic lasting 45-60 seconds weekly. Anti-epileptic treatments included ketogenic diet, clobazam and topiramate. She was not noted to be hypotonic but did have spasticity. Brain MRI findings revealed modest bilateral increased signal in a periventricular distribution, a little more prominent on the left around the trigone and also in the cerebellar peduncles. There was also modest bilateral periventricular leucomalcia. She was not noted to be dysmorphic.

She was recruited to the 100KGP and the primary analysis did not identify any pathogenic variant. Our cohort analysis revealed a homozygous n.102T>C variant in RNU2-2.

Sibling (Individual 37)

This is the brother of P36. Clinical symptoms were first noted at 5 months. Pregnancy and perinatal history were unremarkable. Delivered at term weighing 4.08kg. Growth assessment at age 8 years: weight measured 26kg and OFC was 50.5 (-1.30).

Developmental history was notable for motor delay. He also was unable to roll over and never sat. He has no words and has profound intellectual disability.

He has seizures which are challenging. Ambulatory EEG was globally slowed with multifocal discharges and runs of slow spike wave. Overall, the recording demonstrated an epileptic encephalopathy with myoclonic, tonic and tonic-clonic seizures captured. The EEG pattern would meet criteria for Lennox-Gastaut syndrome. Current seizure burden is around 2 per week maximum and minimum one per month. They last around 45 seconds. He has tonic posturing of the legs and arms, eyes open wide. Anti-epileptic treatments include ketogenic diet, clobazam, epilim and previously levetiracetam and lamotrigine. He does not have hypotonia but does have spasticity. He has upslanted palpebral fissures.

Given the similar presentation to his sister, targeted Sanger sequencing was performed confirming homozygosity for the n.102T>C variant in RNU2-2.

Family 6 (n.104T>C)

Proband (Individual 7)

This is a male individual of Somali background, currently aged 16y 7m. He is the brother of P8 and P9. Clinical symptoms were first noted at 12 months of life. Pregnancy and perinatal history were mostly unremarkable but he was small for dates. Delivered at 40 weeks. Birth weight was 3.4kg. Birth OFC was 34cm.

Growth assessment: at age 16y 7m years, height measured 167cm (9th-25th centile). weight measured 45kg (0.4th-2nd centile) and OFC was 54 (2nd-9th centile).

Developmental history was notable for motor delay. He rolled over at 8 months. Sat unsupported at 12 months. First steps were at 21 months. He had language delay with first words at 24 months. He is mainly non verbal, but says some single words and tends to repeat words. He has severe intellectual disability. Can use You Tube. Can write letters and numbers, uses PECS and recognises words but can't read texts. He has a diagnosis of Autistic Spectrum Disorder.

Seizures began at 33 months of age. Generalised tonic-clonic seizures and anti-epileptic treatments include sodium valproate. However, seizures improved and he stopped treatment age 5 years. He was hypotonic without spasticity. Brain MRI was reported as normal. He has prominent upper incisors but no other dysmorphic features. He has mild to moderate sensorineural hearing loss.

He was recruited to the 100KGP and the primary analysis did not identify any pathogenic variant. Our cohort analysis revealed a homozygous n.104T>C variant in RNU2-2. Additional genetic findings included: 431kb amplification 15q26.3region inherited from father. Hg19 arr (XY) x1,15q26.3(100,192,999-100,623,915)x3 pat.

Sibling (Individual 8)

This is a female individual of Somali background, currently aged 19y 11m and the sister of P7 and P9. Clinical symptoms were first noted at 2 years. Pregnancy and perinatal history were normal. Delivered at 40 weeks with birth weight was 2.8kg.

Growth assessment: at age 19y 11m years, height measured 174.5cm (91st-98th centile). weight measured 76.2kg (91st-98th centile). OFC was 56.5cm (75th-91st centile).

Mild motor delay was not. She sat unsupported at 12 months. First steps at 18 months. Her first words at 48m and can speak in sentences. She has intellectual disability but attends mainstream with 1:1 until year 6. Then at a school for moderate learning disability, can read at about level of a 6 year old but can't add numbers. Able to watch and find things on You Tube. She has a diagnosis of Autistic Spectrum Disorder.

She has not had seizures and an MRI brain has not been performed. She also has prominent upper incisors. She was also recruited to 100KGP and our cohort analysis identified the n.104T>C variant in RNU2-2 in homozygosity.

Sibling (Individual 9)

This is a female individual of Somali background, currently aged 25y 9m, She is the sister of P7 and P8. Clinical symptoms were first noted at 2 years. Pregnancy and perinatal history were mostly unremarkable although she required oxygen at birth. Delivered at 40 weeks with birth weight 3kg.

Growth assessment at age 25y 9m years, height measured 164cm (50th centile). weight measured 72.5kg (91st-98th centile) and OFC was 56cm (50th-75th centile). She also had motor delay. She sat unsupported at 18 months and took her first steps at 30 months. Her first words at 30m and she can now speak in sentences but in general communicates non verbally. She has severe intellectual disability: She is unable to read or write, does not engage or use phone at all. She also has a diagnosis of Autism spectrum disorder.

Seizures began at 14 years of age with generalised tonic-clonic semiology and now occur every 4 weeks approximately. Anti-epileptic treatments included Lamotrigine 150mg bd. She has hypotonia without spasticity. Brain MRI findings were normal aged 14 years.

She was also recruited to 100KGP and cohort analysis identified the n.104T>C variant in RNU2-2 in homozygosity.

Family 7 (n.116_127del)

Individual 10

This is a male individual of South Asian background, currently aged 14 years. Clinical symptoms were first noted at 6 months. Pregnancy and perinatal history were unremarkable. Delivered at 38+6 weeks with birth weight 7lb1oz.

Growth assessment at age 14y years, OFC was 51.5cm (-2.42 SD). He had severe global developmental delay and has never achieved sitting or talking.

Seizures began at 6 months with eye rolling and tonic-clonic nature. Followed by daily seizures for a week whilst inpatient. Initially, he had no response to Keppra and Topiramate. Tonic-clonic seizures persisted until 3-4 years and then stopped. Seizures improved when he stopped topiramate. He now has absences and has episodes of lost of tone lasting a few seconds. He remains on Keppra. He has hypotonia without spasticity. MRI scan at 2 years revealed enlarged CSF spaces in middle cranial fossa bilaterally. Increased CSF spaces bifrontally also. A follow up scan at 4 years suggested fronto-temporal atrophy. His EEG revealed multi-focal epileptiform discharges. He has microcephaly with open-mouthed appearance. He has been PEG fed since 4 yrs. He developed dystonia with rhythmic arm movements aged 4 years and has trial.

He was recruited to the 100KGP and the primary analysis did not identify any pathogenic variant. Our cohort analysis revealed a homozygous n.116_127del variant in RNU2-2. Other genetic findings included a homozygous c.4021+3A>G variant in ATP7B (chr13:g.51937273T>C), however his 24 hour urine copper as well as serum copper and caeruloplasmin were within normal limits.

Family 8 (n.127G>C)

Individual 11

This is a male individual of South Asian background, currently aged 19 years. Pregnancy and perinatal history were unremarkable. Delivered at 41+4 weeks with birth weight 8lb7oz. Growth assessment at age 19y years, OFC was 57.2cm (+1.46).

He had motor delay and sat unsupported at 8-9 months. His first steps at 5 years for 3 weeks only and then stopped. He is now wheelchair bound. He did initially babble but never attained any words. He has severe intellectual disability.

Seizures began at 9 months associated with a fever. He then had a 3 week admission with difficult to control seizures. He had generalized tonic-clonic seizures without fever from around 18 months. Seizures were initially controlled with phenytoin. Seizures returned again around 10-11 years and he was started on Epilim. A GTCS requiring ITU admission occured aged 17 years and Keppra started. He has hypotonia without spasticity. His brain MRI was reported as normal. no. He has a double hair-whorl with hirsute limbs and a café-au-lait spot on the left leg. He also has a hypopigmented mark on the right buttock.

Family history was notable for a sister affected by trichothiodystrophy likely due to a homozygous GTF2H5 c.116A>T (p.Asp39Val) variant. She is not affected by epilepsy and is able to walk and talk. Genetic analysis revealed homozygosity for n.127G>C in RNU2-2. His sister was homozygous wild-type. Additional genetic findings included a homozygous SLC25A22 c.190G>A (p.Gly64Ser)) variant and 15q11.2 microdeletion (BP1-BP2). The microdeletion is a neurosusceptibility locus, however would not explain his severe epileptic presentation. Biallelic pathogenic variants in SLC25A22 are associated with developmental and epileptic encephalopathy type 3 (OMIM #609304) and the gene is also located on chromosome 11 liked RNU2-2. This SLC25A22 missense variant remains a variant of unknown significance which may or may not be contributing to the phenotype observed in Individual 11.

Family 3 (n.166G>C)

Individual 3

This is a female individual of South Asian background, currently aged 30y. Clinical symptoms were first noted at 4 weeks. Pregnancy and perinatal history was unremarkable. She was delivered at 41 weeks and birth weight was 5lbs10oz. Birth OFC was 32.5cm (-1.56). Growth assessment at age 30y years, OFC was 47.8cm (-6.15).

She had motor delay. She rolled over and sat unsupported at 3 years. She never achieved ambulation. She is non-verbal and has severe intellectual disability.

Seizures began at 4 weeks with generalised tonic semiology. Weekly initially, then less often after 2 years. Last seizure was over 10yrs ago. EEG was within normal limits.. Anti-epileptic treatments include Epilim 300mg BD, previously phenobarbitone. She has axial hypotonia and leg spasticity. Brain MRI findings at 3 months revaled normal myelination, , normal grey matter differentiation, with mild degree of cerebral hypo-development. She has microcephaly with low handing columella and open-mouthed appearance. Parents feed and water with pureed diet.

Genetic analysis revealed homozygosity for n.166G>C in RNU2-2. Additional genetic findings included: TMX2 c.406G>A (p.Glu136Lys) homozygous. Biallelic variants in TMX2 have been associated with a neurodevelopmental disorder with lissencephaly (OMIM #618730)[1] however she does not have lissencephaly and all reported cases have carried the same c.500G>A which has been shown to have arisen independently in different populations. This TMX2 variant remains a variant of unknown significance which may or may not be contributing to Individual 3's phenotype.

Family 21

Family 21 are maternal half siblings with complex refractory epilepsy and severe intellectual difficulties. There is limited parental information available, but their mother had learning difficulties and childhood epilepsy. Their maternal grandmother had a hyperkinetic movement disorder and epilepsy.

Proband (Individual 27, n.5C>T | n.129T>C)

This is a female individual of White British background, who passed away at the age of 16y. She was adopted at 18 months of age. She developed what appeared to be myoclonic absence seizures at around 11 months of age. Initially this presented with 'headbutting' (head drops). She developed multiple seizure types (myoclonic seizures, absence seizures, focal seizures, and frequent full body convulsive seizures). The tonic-clonic seizures became more frequent with age. She developed severe intellectual disability (communicated with a few single words), with no regression. She displayed challenging and autistic behaviours. She had a mildly ataxic gait but no other movement disorder, and her motor development was normal. There was no deterioration in functioning over time to suggest progressive myoclonic epilepsy. Her MRI brain at age 4 revealed subtle loss of grey matter differentiation at the left temporal pole with subtle increased grey area in cortex, possibly seizure related. No other obvious epileptogenic focus was identified, and there with no other MRI features of a neurometabolic disorder. Sadly, she passed away from an epilepsy related accident at the age of 16.

Sibling (Individual 28, n.5C>T | n.*9_*29del)

This is a male individual of White British background, currently aged 17y. He has severe drug resistant encephalopathic epilepsy, a florid hyperkinetic movement disorder with severe motor disorder (GMFCS V), severe intellectual disability, severe microcephaly (-5SD), severe gut dysmotility, poor weight gain, grade 4 nephrocalcinosis and urinary retention.

He was adopted into the care of his family member with his sister at birth. He cried inconsolably in the first week of life and his oxygen levels were noticed to drop during sleep. His early milestones were normal. He smiled, sat up, babbled and his tone was reportedly normal. At 6 months of age, he developed infantile spasms. He was unable to roll, sit or babble and did not gain new skills. His seizures evolved to include tonic-clonic seizures and absence seizures, requiring multiple antiepileptic medications. He developed a florid movement disorder not associated with spasticity or dystonia, which was improved with tetrabenazine and deteriorated with clonidine. Due to severe gut dysmotility he required jejenal feeds s to gain weight. He has had several intensive care admissions with respiratory infections and secretion related problems. MRI brain revealed progressive supra and infratentorial white matter volume loss with several T2 hyperintensities in the right frontal lobe, hazy hyperintensity in posterior parietal white matter and atrophy of the cerebellar folia and vermis.

In the sister, we identified compound heterozygous *RNU2-2* variants (n.5C>T and n.129T>C). However, we only observed the maternally-inherited n.5C>T variant in the half-brother. Given that variants in the 3' extension of other snRNAs are known to be pathogenic (e.g. RNU12[2] and SNORD118[3]), we searched for variants downstream of *RNU2-2*, and identified a 20bp deletion (n.*9_*29del) *in trans* with n.5C>T.

Family 14 (n.8C>T | n.104T>G)

Individual 17

This 30-year-old White British woman was born at 41 weeks following an unremarkable pregnancy and spontaneous delivery. Birth weight was 3203g. Immediately after birth, she developed breathing difficulties thought to be due to swallowed mucus. Despite neonatal respiratory symptoms, there were no cardiac or structural anomalies documented.

By nine months of age she demonstrated clear developmental delay, affecting gross motor skills and early communication. She walked at 30 months unaided and continues to be ambulant. Throughout childhood she exhibited persistent global developmental delay and later required specialised educational support. She has severe intellectual disability with concomitant ADHD. She is non-verbal.

Growth parameters showed short stature and microcephaly in childhood. Musculoskeletal findings included scoliosis and bilateral flexible pes planus, contributing to periods of pain and reduced mobility.

Her first seizure at 2.5 years (eyes flickering and lips smacking with arms and legs tonic and clonic activity) occurred when she had an ear infection and this was thought to be a febrile seizure. Aged 4.5 developed weekly episodes of subtle eye rolling and gentle twitching lasting a few seconds. At this time, she would not lose tone and would not fall. Initially managed with valproate but re-occurred within 1 year. In total, she has had 4 admissions to hospital with prolonged seizures. Also had clinical absences and myoclonus but these do not occur anymore after age 12 years.

EEG aged 13 years was diffusely abnormal with severe slowing, frequent sharp and delta activity with epileptiform discharges. Multiple stereotyped events of flinching, arms moving up, rocks back and forth, looking frightened and gasping. EEG aged 27 showed some episodes similar. Serial MRI scans of her brain were unremarkable aged 4 and 10 years.

Genetic evaluation revealed compound heterozygous variants in RNU2-2: n.8C>T and n.104T>G. As she was recruited to 100KGP with her mother, the n.8C>T variant was known to be maternally inherited. Read-backed phasing confirmed the n.104T>G variant to be *in trans* in the absence of a paternal sample. All additional genetic investigations, including array CGH and whole-genome sequencing, yielded no further pathogenic findings. There is also no relevant family history.

In adulthood, she developed osteoporosis complicated by a hip fracture. Current medications include depo-provera and colecalciferol. She lives with chronic functional impairment secondary to neurodevelopmental disability but no major systemic health issues.

Family 47 (n.12G>C | n.171T>C)

Individual 57

This 13-year-old Korean boy first came to attention of clinical genetics at 9 years and 3 months with concerns regarding developmental regression and seizures.

He was born at term after an uncomplicated pregnancy. His early development was unremarkable and he walked between 12 and 13 months. He experience motor regression after his first seizure at around 5 years and now has a unsteady gait. He was initially able to use 2-3 word sentences but these regressed and he now is non-verbal. He has moderate intellectual disability. He does not have autistic features or hyperactivity. He does, however, experience ataxia and tremor.

Since the age of 5 years, he has experience intractable seizures of multiple semiology. Valproic acid, oxcarbazepine and dietary restriction were of no clinical benefit. His MRI scan was unremarkable.

Analysis of the South Korean Undiagnosed Diseases dataset identified compound heterozygous variants in RNU2-2: n.12G>C and n.171T>C. No alternative diagnoses were identified through whole genome sequencing.

Family 41 (n.18_21delinsGGG | n.45C>T)

Individual 50

This is a male individual of White British background, currently aged 26 years. He was born at term and weight 7lb 7oz. He was a snuffly baby and feeding was challenging in the neonatal period.

At 22 months, his weight was 10.8kg, height was 87cm and OFC was 47.9cm.

He smiled at 6 weeks but presented as a hypotonic baby at 11 months of life. At that time, he was able to roll but by 18 months, he was able to stand but was unsteady. His fine motor movements were also very jerky.

EEG showed high voltage paroxysm of irregular spike and wave and poly spike and wave complexes. He developed seizures at which are generalised. Myoclonic seizures. He developed choreoathetoid movements aged 9 years and was trialled on tetrabenzanine.

He had targeted testing for Angelman syndrome, microarray and whole exome sequencing as part of the Deciphering Developmental Disorders (DDD) Study without a diagnosis found. Whole genome sequencing was undertaken as part of the UK Genomic Medicine Service which did not identify any pathogenic variants (PanelApp panels R29 and R59). Cohort wide reanalysis of the GMS data in NGRL revealed compound heterozygosity for (n.18_21delinsGGG | n.45C>T) in RNU2-2.

Family 35 (n.19G>A | n.52G>A)

Individual 44

This is a male individual of White British background, currently aged 14. Clinical symptoms were first noted at 6 months. Pregnancy and perinatal history were unremarkable. Ventouse delivery. Required oxygen briefly at birth but no SCBU admission. Delivered at 41+6 weeks. Birth weight was 4.762kg (+2.55SD).

He had motor delay and rolled over at 13 months. Sat unsupported at 13 months. First steps at 24 months. He has speech delay and is now non-verbal. He does make sounds but no words. He has severe intellectual disability and diagnoses of Autistic Spectrum Disorder and Attention Deficit Hyperactivity Disorder.

He has a history of drug-refractory epilepsy and has episodes in time where the seizures are more frequent. He has a vagal nerve stimulator and takes sodium valproate and clobazam. He has hypotonia and spasticity in the legs requiring baclofen. MRI scans revealed a left cranial cyst and cerebral atrophy. He has fleshy ear lobes, wide gap between toes but not otherwise dysmorphic.

Genetic analysis revealed compound heterozygosity for (n.52G>A | n.19G>A) in RNU2-2.

Family 27 (n.19G>A | n.63G>A)

Individual 34

This is a female individual of White British background is currently aged 34 years. Clinical symptoms were first noted at 7 months. Pregnancy and perinatal history were unremarkable. Delivered at 41 weeks. Birth weight was 6lbs 7oz. Growth assessment at age 33 years 8 months, height measured 156cm (13th centile), weight measured 59.9kg (50th centile) and OFC was 57cm (+2.42SD).

She sat unsupported between 1.5-2 years. She has never walked and vocalizes with repetitive sounds but not coherent words. She has severe intellectual disability.

Absences were noted aged 6 months-8 years, from age 8-12 years she had 30-40 drop attacks a day. When older, she developed generalised tonic-clonic seizures once a week. In summary, she has complex epilepsy with drop attacks, myoclonic jerks, absences, tonic clonic seizures. Anti-epileptic treatments include Epilim 500mg OM 700mg ON, clobazam 15mg ON, levetiracetam 1000mg BD, zonisamide 150mg OM 200mg ON. She is not hypotonic and does not have spasticity. Brain MRI findings are significant for white matter loss, thin corpus callosum and cerebellar atrophy. She has thickened lips, a wide mouth , short philtrum and prominent forehead.

Genetic analysis revealed compound heterozygosity for (n.19G>A | n.63G>A) in RNU2-2.

Family 49 (n.20G>A |n.21C>A)

Individual 59

This Korean girl, now 8 years and 10 months old, was born a term following an uncomplicated pregnancy. She has demonstrated motor delay and currently is able to sit unsupported. She has never walked. She has severe developmental delay and absent speech.

Her seizures began aged 10-12 months with staring spells and motion arrest. She has been treated with valproate, zonisamide, levetiracetam and perampanel but with little clinical benefit. She has truncal hypotonia, microcephaly and has developed a hyperkinetic movement disorder with hand stereotypy. Her brain MRI scan was unremarkable

Analysis of the South Korean Undiagnosed Diseases dataset identified compound heterozygous variants in RNU2-2: n.20G>A and n.21C>A. No alternative diagnoses were identified through whole genome sequencing.

Family 48 (n.21C>A | n.180G>A)

Individual 58

This 8-year-8-month-old Korean girl was born at term following and uncomplicated pregnancy. She had demonstrated global developmental delay, attaining her first steps at 4 years with a wide-based gait. She walked for only one year and then experienced regression and is now unable to walk independently. She has never attained speech and has severe intellectual disability.

She has severe sleep disturbance with hand stereotypy. She has bruxism, predominantly on waking and abnormal vocalisations. Her seizures began at 4.5 years with staring episodes following by drop attacks. Seizures have been relatively unresponsive to valproate and levetiracetam. She has truncal hypotonia and is normocephalic. Her brain MRI scan is unremarkable. She has a facial appearance reminiscent of Angleman syndrome and pale skin.

Analysis of the South Korean Undiagnosed Diseases dataset identified compound heterozygous variants in RNU2-2: n.21C>A and n.180G>A. No alternative diagnoses were identified through whole genome sequencing.

Family 28 (n.25G>A | n.131G>C)

Individual 35

This is a female individual of White British background, currently aged 11ears 5months. Clinical symptoms were first noted at 7 months. Pregnancy and perinatal history were unremarkable. Delivered at term plus 15 days. Birth weight was 3kg. Growth assessment: at age 11y5m years, height measured 151.1cm (75th centile). weight measured 40.7kg (50th-75th centile).

Motor delay was reported. She rolled over at 14 months and sat unsupported at 12-14 months. First steps at 20 months but very wobbly. Independent walking delayed until over 2years of age. Speech/language delay but always sociable, smiling, sentences age 4. Intellectual disability is present and she attends specialist setting. She has some autistic behaviours but does not fulfil criteria for a diagnosis.

Focal epilepsy presented with left sided facial weakness, slurred speech and drooling aged 8 years. Anti-epileptic treatments include Carbamazepine 14mls BD (280mg BD), Lamotrigine 150mg BD, Clobazam 10mg at nightime. Her tone was normal and brain MRI reported as normal. She is non-dysmorphic.

Family history notable for 16p13.11 deletion, a neurodevelopmental disorder susceptibility locus, which is paternally inherited. A pathogenic TRIO variant Chr5:g.14487936del associated with intellectual development disorder-44 with microcephaly was also maternally inherited. Genetic analysis revealed compound heterozygosity for (n.25G>A | n.131G>C) in RNU2-2.

Family 17 (n.28G>C | n.158G>C)

Proband (Individual 20)

This is a female individual of White British background who died at 23 years. Pregnancy and perinatal history were normal. Delivered at term weighing 8lbs. OFC in adulthood was 54 cm (-0.29).

She did not have motor delay and sat unsupported at 8 months. First steps at 15 months. She never attained words and had severe intellectual disability with a diagnosis of Autistic Spectrum Disorder.

Seizures were noted shortly after birth. These were photosensitive, generalised tonic-clonic and tonic in semiology. Anti-epileptic treatments included valproate, lamotrigine, zonisamide. She had no tone abnormalities. She had deep set eyes and low hanging columella.

She was recruited to 100KGP along with her similarly affected brother but no plausible diagnostic variants were reported. Further analysis revealed compound heterozygosity for (n.158G>C | n.28G>C) in RNU2-2.

Sibling (Individual 21)

This is a male individual, sibling of P20. Pregnancy and perinatal history was normal. Delivered at Term weeks. Birth weight was ~7lbs. OFC was 58cm in adulthood.

He had motor delay. First steps at 24 months. He has never attained words and has severe intellectual disability.

He has myoclonic, generalised tonic-clonic and focal seizures. Anti-epileptic treatments include valproate, lamotrigine, perampanel, clobazam, clobazam, vigabatrin, topiramate, ethosuximide. He has facial hypotonia without spasticity. Brain MRI was reportedly normal in childhood. EEG showed bifrontal epileptiform changes. He has sloping forehead, facial hypotonia and protruding tongue.

Like his sister, we identified compound heterozygous (n.158G>C | n.28G>C) RNU2-2 variants on re-analysis of the 100KGP data.

Family 46 (n.157G>C | n.31G>A)

Individual 56

This 14-year-3-month-old Korean boy first presented at 27 months with global developmental delay. Delays were most pronounced in expressive language, social communication, and fine motor coordination. He walked at 27 months, around the time of his referral. Over time he has exhibited academic difficulties requiring ongoing support, with features suggestive of inattention and autism-spectrum traits. He has demonstrated mild speech delay.

He has partial seizures only and these have been well-controlled with valproate. His MRI brain scan was unremarkable and he has no other neurological features.

Notably, whole genome sequencing identified a de novo missense variant in MTOR (c.4283C>T, p. Ala1428Val). He has mild to moderate intellectual disability with macrocepahly (95th centile), partially matching Smith- Kingsmore syndrome

Family 36 ([n.36G>A ;n.154C>T] | n.182T>C)

Individual 45

This is a female individual of White British background, currently aged 5 yrs. Clinical symptoms were first noted at 2yrs 11 months. Pregnancy and perinatal history were unremarkable. Delivered at term weighing 3.06kg. Growth assessment at age 2yr 11m years, height measured 87.3 cm (-1.87 SD). weight measured 10.6 kg (-2.06 SD). OFC was 46.5 cm (-1.36 SD).

She rolled over at 3yrs 3 months and sat unsupported at 12 months. She has not walked independently. Speech/language delay was severe and she is now non-verbal. She has severe intellectual disability with autistic behaviour.

She has not developed seizures. Her MRI brain was reported as normal at 1 year of age. She has a bulbous nasal tip, thin upper lip, slightly up slanting palpable fissures, prominent ears and synophrys.

Cohort wide re-analysis of the 100KGP data revealed compound heterozygous RNU2-2 variants ([n.182T>C] | [n.154C>T;n.36G>A]).

Family 26 ([n.43T>G;n.62T>G] | [n.162dup;n.189T>C])

Individual 33

This is a male individual of White British father and Asian mother, currently aged 19. Clinical symptoms were first noted at 19 months. Pregnancy and perinatal history were unremarkable. Delivered at 38 weeks by emergency LSCS, weighing 3.92kg. Growth assessment at 2 years, height measured 91st centile, weight measured 75th centile and OFC was 49.5 cm (25th).

He rolled over at 24 months and sat unsupported at 9 months. First steps at 25 months. Walked with side-based gait, frequent falls. He initially had a few words then regressed to non-verbal. He has severe intellectual disability.

He started with seizures at 27 months which were very difficult to control and associated with regression of skills. Absence seizures age 27 months, drop seizures age 41 months, now aged 19 years, myoclonic and tonic seizures. Anti-epileptic treatments include sodium valproate but developed peeling of skin then topiramate and clobazam and then levetiracetam then Rufinamide age 4 years. Currently, aged 19 years on rufinamide, clobazam, cannabidiol trial and lamotrigine. He has hypotonia and intermittent dystonic posturing especially on the right side. Brain MRI was reported as normal at 27 months. He has periorbital fullness, open mouth and full lower lip. He has 3-4 café au lait macules >0.5 cm and a skin tag medial to left eye.

Cohort wide re-analysis of the 100KGP data revealed compound heterozygous RNU2-2 variants ([n.189T>C;n.162dup] | [n.62T>G;n.43T>G]).

Family 22 (n.43T>C | n.175G>A)

Individual 29

This is a female individual of Romanian background, currently aged 16y. Clinical symptoms were first noted at 6 months. Pregnancy and perinatal history was unremarkable. Delivered at 39 weeks by Caesarean section in good condition. Birth weight was ~3kg. Growth assessment at age 16 years, OFC was 52cm (-2.16).

Her motor development was delayed and she rolled over at 2 years, sat unsupported at 18m months, took first steps at 8 years. She has never attained words and has severe intellectual disability.

She has multiple seizure types and anti-epileptic treatments include lamotrigine, valproate, and keppra. She has hypotonia without spasticity. Brain MRI suggested some asymmetry with possible volume loss and poorer grey-white matter differentiation on the right. She has microcephaly, slight upslanting palpebral fissures and thick lips.

She was recruited to 100KGP but no pathogenic variants were identified on primary analysis. Cohort-wide re-analysis identified compound heterozygous RNU2-2 variants (n.175G>A | n.43T>C).

Family 32 (n.45C>G | n.50C>T)

Individual 41

This is a female individual of White British background, currently aged 7 years. Clinical symptoms were first noted at 6 months. Pregnancy and perinatal history was notable for a small head on the 20 week anomaly scan. Her OFC has tracked 3rd centile since birth.

She has motor delay and has not achieved ambulation. She also has severe intellectual disability with no words.

She has seizures which began aged 7 years which occur the same week of every month. Her EEG shows irregular sharp slow wave complexes which vary in emphasis between the cerebral hemispheres in sleep, occasionally appearing more diffuse. The wake recording was unremarkable. Anti-epileptic treatment is currently being considered but no yet started. She has hypotonia without spasticity but display episodic stiffening with dystonic posturing. Brain MRI showed Arnold Chiari malformation with absent septum pellucidum. She has very subtle epicanthic folds with a broad nasal root.

She had WGS as part of the UK Genomic Medicine Service without any diagnostic results found. Subsequent re-analysis targeted at biallelic RNU2-2 variants in the cohort identified compound heterozygous (n.50C>T | n.45C>G) variants.

Family 34 (n.45C>T | [n.67C>T;n.183_184insA])

Individual 43

This 14-year-old British boy was born at 42 weeks following an uncomplicated pregnancy. He has displayed normal motor and language milestones but has intellectual disability. His current learning age is 5.5 years. He is treated with methylphenidate for ADHD and requires melatonin for sleep.

He has seizures which occur on a monthly basis. His EEG shows irregular sharp slow waves in sleep only. He has not yet started any medication as seizures have only just started.

His MRI brain was remarkable for an Arnold-Chiari malformation and absent septum pellucidum. He does display some dystonic posturing.

Cohort-wide analysis of the Genomic Medicine Service data within the NGRL identified compound heterozygous variants in RNU2-2: n.45C>T and a complex haplotype (n.67C>T ; n.183_184insA). No other variants were identified through whole genome sequencing to explain his phenotype.

Family 42 ([n.56A>T ;n.157G>C] | n.104T>C)

Individual 51

This is a female individual of English/Irish background, currently aged 5 years 3 months. Clinical symptoms were first noted at 6 months. Pregnancy and perinatal history was unremarkable. She was born at 38/40 weeks, weighing 2.86kg. Her birth OFC was 34cm. Growth assessment: at age 4 years 9 months years, height measured 100.00cm (-0.49). weight measured 18.7kg (0.54) and OFC was 50.5 (0.52).

She has demonstrated motor delay. She rolled over at 8 months and sat unsupported at 6 months. Her first steps were not taken until 26 months. She has severe-profound intellectual disability with a diagnosis of Autism spectrum disorder and is non-verbal.

She has myoclonic epilepsy of infancy/childhood. Anti-epileptic treatments include Clobazam and Sodium Valproate but she was previously taking Levetiracitam (initial good response but then ineffective) and Zonisamide (developmental regression/choking). She has brisk reflexes but no spasticity or hypotonia. Her MRI brain was reported as normal. She does not appear dysmorphic.

Targeted analysis of the UDNAus dataset for biallelic RNU2-2 variants identified compound heterozygosity ([n.56A>T | n.157G>C] | n.104T>C). She has otherwise no plausible candidate variants to explain her neurodevelopmental disorder.

Family 44 (n.104T>C | n.100T>G)

Individual 53

This is a female individual of White European background, currently aged 13 years. Clinical symptoms were first noted at 9 months. Pregnancy and perinatal history was unremarkable. She was born at 36 weeks, weighing 3kg. Her birth OFC was 33.5cm. Growth assessment: at age 7 years, height measured 130cm, weight measured 36kg and OFC was 50.5cm.

She has demonstrated motor delay. She rolled over at 5 months but has never sat unsupported or gain ambulation. She has absent speech although did babble from 6 months of age. She has severe intellectual disability.

She has tonic-clonic seizures and takes Depakin 200 (frisium 10mg – Epidolex 3,5 ml). She does not have hypotonia but does have spasticity on examination. Brain MRI revealed a modest increase in the representation of the CSF space of the cisterns of the left cerebellopontine angle with cerebellar hypoplasia. She does not appear dysmorphic.

WGS was performed as part of SOLVE-RD and no plausible diagnostic variants were returned. Targeted analysis of the cohort data through GPAP for biallelic RNU2-2 variants identified compound heterozygosity (n.104T>C | n.100T>G).

Family 45 (n.104T>C | n.159_176dup)

Proband (Individual 54)

This is a female individual of White European background, currently aged 15 years. Clinical symptoms were first noted at 6-7 months. Pregnancy and perinatal history were unremarkable. She was born at term weighing 3kg. She was noted to be microcephalic at birth. Growth assessment at age 2 years, height measured 134cm, weight measured 20kg and OFC was 49cm.

She has demonstrated motor delay. She rolled over at 5 months but has never sat unsupported or achieved ambulation. She has absent speech with severe intellectual disability.

Seizures started at 6 months of age, which were predominantly myoclonic in semiology. Anti-epileptic treatments include Keppra 50mg x 2. On examination, she has limb spasticity without central hypotonia. She has frontal bossing, sunken eyes and slight prognathism.

WGS was performed as part of SOLVE-RD and no plausible diagnostic variants were returned. Targeted analysis of the cohort data through GPAP for biallelic RNU2-2 variants identified compound heterozygosity change (n.104T>C | n.159_176dup).

Sibling (Individual 55)

This is a male individual of White European background, currently aged 14 years and the younger brother of P54. Clinical symptoms were first noted at 10 months. Pregnancy and perinatal history was unremarkable. He was born at term weighing 3.5kg. Birth OFC was normal. Growth assessment at 14 years, height measured 140cm, weight measured 24.5kg and OFC was 48.5cm.

He displayed motor delay. He rolled over at 5 months and sat unsupported at 6 months. He has, however, never achieved ambulation. He has absent speech and severe intellectual disability.

Seizures and tremor started at 10 months of age. For seizures, he takes Keppra. On examination, he has mild limb spasticity but no hypotonia.

Segregation analysis for the n.104T>C | n.159_176dup RNU2-2 variants originally identified in his sister showed that he was also compound heterozygous for this variant combination.

Specific variants and variant combinations

We have previously shown that heterozygous variation in the 5' end of RNU2-2 (n.1 to n.67) is highly constrained in gnomAD v4.1[4]. This region contains nucleotides which carry extensive post-transcriptional modifications and are involved in inter-molecular base pairing with U6 and the splicing branchpoint.

Distinct from the dominant *RNU2-2* syndrome, where variants are recurrent, we observed a diverse set of variant combinations in affected cases in this study. All of these variants are rare in gnomAD, however, we do observe some recurrent variants in unrelated families.
Four variants were observed in both the homozygous and compound heterozygous states (n.20G>A, n.28C>G, n.104T>C and n.127G>C). Variant positions in unsolved NDD cases were distributed throughout the primary sequence of *RNU2-2*, apart from the unstructured sequence between stem loop IIb and the Sm binding site (n.85 to n.96) which was devoid of variants in these individuals (Fisher's exact P=0.0091). Variants in unsolved NDD cases overlapped nucleotides important for snRNA-snRNA interactions between U2 and U6, snRNA-protein interactions within the Sm protein binding site, and U2 secondary loop structures including stem loops IIa, IIb, III and IV.

Recurrent variants in unsolved NDD cases were n.45C>T and n.104T>C, which were each observed in two and three unrelated families, respectively. Different substitutions were observed at these nucleotides also (n.45C>G and n.104T>G) in the cohort. The recurrence of variants in affected individuals, and the occurrence of variants at positions which base-pair in the snRNA secondary structure, provide supporting evidence of pathogenicity. For example, three individuals from two unrelated families (Families 4 and 5), all of Pakistani descent, carried the same n.20G>A variant in homozygous state. Haplotype analysis revealed a shared region of homozygosity of 30Mb between these unrelated families, suggesting a common ancestor. This variant was also observed in another individual, in the compound heterozygous state, *in trans* with n.61C>T (Family 12). n.20 base-pairs with n.13 in the U2-2 secondary structure; we observe n.13C>A_in one individual (Family 9) in the compound heterozygous state, in trans with n.100T>G, within the Sm site. Interestingly, we observed a recurrent indel in both the homozygous and compound heterozygous state in three unrelated individuals (n.116_127del in families 7 and 40, and n.116_127dup in family 23). We observed four additional indels in unsolved NDD cases. All were in the compound heterozygous state, and all were located in the 3' end of *RNU2-2*, downstream of n.115. Two of these (n.170_*5del and n.186_*27del) overlapped the 3' extension of U2-2 pre-snRNA which is known to be transcribed and cleaved post-transcriptionally, and which contains nucleotides important for regulating transcription termination and stability during nuclear export[5].

Interestingly, we find two variants in the compound heterozygous state (n.3C>T and n.5C>T) which overlap sites at which specific SNVs (n.3C>A and n.5C>A) we had previously linked to the dominant RNU2-2 syndrome[4]. In both cases, the variants in the dominant condition are observed in individuals with milder presentations, have been seen to be transmitted or parental samples were not available to determine de novo origin. In the family carrying the n.5C>T variants, this was inherited by two affected half-siblings from their unaffected mother. Each half-sibling had, in fact, inherited a different variant from each of their unrelated fathers suggesting that the gene carrier rate of this condition may be high.

Variant interpretation is, of course, challenging in a non-coding gene. For individuals with a low U2:U1 transcript ratio, we find evidence for pathogenicity for the following variant combinations: n.41T>A | n.127G>C, n.20G>A | n.61C>T, n.102T>C homozygous, [n.162dup;n.189T>C] | [n.43T>G;n.62T>G], n.183G>C homozygous, n.171_187del | n.186_*27del, n.181G>C homozygous and n.113G>A | n.116_127dup (**Supplementary Table 10)**

Supplementary References

1. Vandervore, L. V. *et al.* TMX2 Is a Crucial Regulator of Cellular Redox State, and Its Dysfunction Causes Severe Brain Developmental Abnormalities. *Am. J. Hum. Genet.* **105**, 1126–1147 (2019).
2. Xing, C. *et al.* Biallelic variants in RNU12 cause CDAGS syndrome. *Hum. Mutat.* **42**, 1042–1052 (2021).
3. Jenkinson, E. M. *et al.* Mutations in SNORD118 cause the cerebral microangiopathy leukoencephalopathy with calcifications and cysts. *Nat. Genet.* **48**, 1185–1192 (2016).
4. Jackson, A. *et al.* Analysis of R-loop forming regions identifies RNU2-2 and RNU5B-1 as neurodevelopmental disorder genes. *Nat. Genet.* **57**, 1362–1366 (2025).
5. Pánek, J. *et al.* The SMN complex drives structural changes in human snRNAs to enable snRNP assembly. *Nat. Commun.* **14**, 6580 (2023).
