# Supplementary material for: Biallelic variants in RNU2-2 cause a remarkably frequent developmental and epileptic encephalopathy
Source: Nat Genet. 2026 Mar 30;58(4):798–809. doi: 10.1038/s41588-026-02551-9 (PMC13083258; doi:10.1038/s41588-026-02551-9)
Supplement: Supplementary file 2 — Reporting Summary [file 41588_2026_2551_MOESM2_ESM.pdf]

nature portfolio

Corresponding author(s): Siddharth Banka

Last updated by author(s): 22/01/2026

Reporting Summary

Nature Portfolio wishes to improve the reproducibility of the work that we publish. This form provides structure for consistency and transparency in reporting. For further information on Nature Portfolio policies, see our Editorial Policies and the Editorial Policy Checklist.

Statistics

For all statistical analyses, confirm that the following items are present in the figure legend, table legend, main text, or Methods section.

n/a	Confirmed	
☐	☒	The exact sample size (*n*) for each experimental group/condition, given as a discrete number and unit of measurement
☐	☒	A statement on whether measurements were taken from distinct samples or whether the same sample was measured repeatedly
☐	☒	The statistical test(s) used AND whether they are one- or two-sided<br>*Only common tests should be described solely by name; describe more complex techniques in the Methods section.*
☐	☒	A description of all covariates tested
☐	☒	A description of any assumptions or corrections, such as tests of normality and adjustment for multiple comparisons
☐	☒	A full description of the statistical parameters including central tendency (e.g. means) or other basic estimates (e.g. regression coefficient) AND variation (e.g. standard deviation) or associated estimates of uncertainty (e.g. confidence intervals)
☐	☒	For null hypothesis testing, the test statistic (e.g. *F*, *t*, *r*) with confidence intervals, effect sizes, degrees of freedom and *P* value noted<br>*Give P values as exact values whenever suitable.*
☒	☐	For Bayesian analysis, information on the choice of priors and Markov chain Monte Carlo settings
☒	☐	For hierarchical and complex designs, identification of the appropriate level for tests and full reporting of outcomes
☐	☒	Estimates of effect sizes (e.g. Cohen's *d*, Pearson's *r*), indicating how they were calculated

Our web collection on statistics for biologists contains articles on many of the points above.

Software and code

Policy information about availability of computer code

Data collection	Data was accessed in the National Genomics Research Library (NGRL) for all individuals in the 100,000 Genomes Project. This included variant data from whole genome sequencing and also Human Phenotype Ontology terms. Recruitment categories and also family relationships and sequencing structures were also derived from this resource. The NGRL also houses the GMS data which was used as a validation cohort in this manuscript.<br><br>Other validation cohorts included Solve-RD, UDNAus, South Korean Undiagnosed Diseases Database, Saudi Arabia Lifera Database and Swedish Undiagnosed Diseases Network, which were accessed through either the RD-CONNECT platform of personal communication with co-authors.
Data analysis	Software used for data analysis are housed within the NGRL and include samtools (v1.10), bftools (v1.16), bowtie2 (v2.5.2), bedtools (v.2.31.0), FRASER2, OUTRIDER.

For manuscripts utilizing custom algorithms or software that are central to the research but not yet described in published literature, software must be made available to editors and reviewers. We strongly encourage code deposition in a community repository (e.g. GitHub). See the Nature Portfolio guidelines for submitting code & software for further information.

Data

Policy information about availability of data

All manuscripts must include a data availability statement. This statement should provide the following information, where applicable:

- Accession codes, unique identifiers, or web links for publicly available datasets
- A description of any restrictions on data availability
- For clinical datasets or third party data, please ensure that the statement adheres to our policy

Genomic and phenotypic data are available for the 100KGP and individuals who have had WGS through the Genomic Medicine Service in the NGRL. Access to the NGRL may be granted following application via https://www.genomicsengland.co.uk/research/academic/join-research-network, which gives access to the secure GERE. Genomic data used pertain to participants in 100KGP in the Main Programme v.18 and the GMS data v.4. SolveRD data are accessible by application through the RD-CONNECT platform. All data presented in this paper, pertaining to 100kGP participants, were requested for the Airlock transfer through GERE. The paper was submitted for approval by the Genomics England Publication Committee on 25th August 2025 and was approved on 27th August 2025. Access to the Australian Centre for Population Genomics dataset can be requested through contact with the authors. The GRCh38 human genome reference assembly can be accessed at https://www.ncbi.nlm.nih.gov/datasets/genome/GCF_000001405.26/. The GENCODE v.32 comprehensive annotations were accessed within the GERE but can be downloaded from https://www.gencodegenes.org/human/release_32.html. The gnomADv4 genotype VCF files were accessed within the GERE but can also be downloaded from https://gnomad.broadinstitute.org/.

Research involving human participants, their data, or biological material

Policy information about studies with human participants or human data. See also policy information about sex, gender (identity/presentation), and sexual orientation and race, ethnicity and racism.

Reporting on sex and gender	Sex is reported for all individuals with prioritized variants, which is founded in their recruitment sex. The disorder occurs with equal prevalence in both sexes and hence sex-specific analyses were not undertaken.
Reporting on race, ethnicity, or other socially relevant groupings	Where available, ethnicity is reported for individuals with detailed phenotype data who have provided additional consent. This refers to their self described ancestry / country / region of origin, rather than race.
Population characteristics	The main analysis of this study was undertaken in NGRL which houses genome sequencing data for over 100 000 individuals who have been recruited as either probands or relatives of probands with a rare disease. These are predominantly children and young adults with respect to neurodevelopmental disorders but we have not stipulated any age cut off in our analysis. Validation cohorts consist of individuals and their families with rare conditions. Not all cohorts stipulated neurodevelopmental disorders as a recruitment criteria although this is a common recruitment category in all.
Recruitment	Participants were prospectively recruited to the 100KGP and the same is the case for the GMS data. Recruitment was led by the Genomic Medicine Services in the UK. Recruitment was prospective for all validation cohorts also.
Ethics oversight	This research was performed under the ethical approvals given by the South Manchester National Health Service (NHS) Research Ethics Committee (REC; 11/H1003/3/AM02). Written informed consent for the inclusion of detailed clinical information, imaging data, and facial photographs, was obtained from all participants or their parents.

Note that full information on the approval of the study protocol must also be provided in the manuscript.

Field-specific reporting

Please select the one below that is the best fit for your research. If you are not sure, read the appropriate sections before making your selection.

☒ Life sciences ☐ Behavioural & social sciences ☐ Ecological, evolutionary & environmental sciences

For a reference copy of the document with all sections, see nature.com/documents/nr-reporting-summary-flat.pdf

Life sciences study design

All studies must disclose on these points even when the disclosure is negative.

Sample size	Sample size was determined by the number of available genomes in the NGRL which is steadily increasing with specific data freezes. No power calculations were performed as the number of cases and controls in the NGRL is finite.
Data exclusions	For trio analyses looking at transmission of RNU2-2 variants, data aligned to GRCh37 was excluded as RNU2-1 locus is not annotated in this genome assembly giving rise to mapping errors which could affect the resultant data and analysis.
Replication	Replication was undertaken by consulting independent rare disease genome sequencing cohorts from around the world. Results were validated in all datasets analysed (GMS, SOLVE-RD, UDNAus, Sweden, Saudi Arabia and South Korea)
Randomization	Randomization was not appropriate as phenotypes needed to be grouped.

Blinding

For statistical analysis, authors were blind to which participants were in the neurodevelopmental disorders group and those who were in the control groups. For phenotype analysis, authors could not be blinded to participant ID and this would be inappropriate for such analyses.

Reporting for specific materials, systems and methods

We require information from authors about some types of materials, experimental systems and methods used in many studies. Here, indicate whether each material, system or method listed is relevant to your study. If you are not sure if a list item applies to your research, read the appropriate section before selecting a response.

Materials & experimental systems

n/a	Involved in the study
☒	☐ Antibodies
☒	☐ Eukaryotic cell lines
☒	☐ Palaeontology and archaeology
☒	☐ Animals and other organisms
☒	☐ Clinical data
☒	☐ Dual use research of concern
☒	☐ Plants

Methods

n/a	Involved in the study
☒	☐ ChIP-seq
☒	☐ Flow cytometry
☒	☐ MRI-based neuroimaging

Plants

Seed stocks

n/a

Novel plant genotypes

n/a

Authentication

n/a
